# No Compliance Correction of Class II Malocclusion in Growing Patients Whit HERBST Appliance: A Case Report

**DOI:** 10.2174/1745017901814010605

**Published:** 2018-09-28

**Authors:** M. Portelli, A. Militi, M. Cicciù, A. Lo Giudice, G. Cervino, R. Fastuca, R. Nucera

**Affiliations:** Department of Biomedical and Dental Science, and of Morphological and Functional Images, Dental School, University of Messina, ‎Messina, Italy

**Keywords:** Skeletal class II malocclusion, Mandibular retrusion, Functional appliance, Mandibular growth, Herbst appliance, No compliance apparatus

## Abstract

**Background:**

Class II malocclusion is the most common sagittal skeletal discrepancy, with a prevalent skeletal pattern of mandibular retrusion. The correction of mandibular retrusion with functional removable appliance needs a good patient’s compliance; for this reason, some clinicians prefer to use no compliance apparatus.

**Objective:**

Objective of the present therapy note is to demonstrate that the use of no compliance apparatus can provide a good correction of skeletal class II malocclusion.

**Methods:**

In the present study, authors report a therapy note referred to a 10 years old patient, woman, affected by Class II, with mandibular retrusion and deep bite, treated in 2013 at the Dep. of Orthodontics of Messina University. An orthodontic treatment has been planned with the aim of stimulating mandibular growth; an Herbst appliance with a cantilever design, bonded on first maxillary and mandibular molars, has been used. After eleven months of functional therapy a bilateral molar class I have been obtained.

**Results:**

In the therapy note proposed, authors obtained a resolution of mandibular retrusion, a correction of overjet, overbite and dental crowding in both arches, and a bilateral molar and canine class I has been achieved.

**Conclusion:**

Herbst appliance seems to be efficient in the correction of II Class Malocclusion, independently from patient’s cooperation; moreover , early correction of Class II malocclusion with functional appliances produces several clinical advantages.

## 1 INTRODUCTION

Class II malocclusion is the most prevalent sagittal skeletal discrepancy [1, 2]. Different skeletal pattern can participate in the development of a Class II malocclusion [3]: mandibular retrusion, sagittal maxillary hyperplasia, or a posterior position of the glenoid fossa [4, 5]. The most frequent aetiologic factor in skeletal Class II malocclusion is mandibular retrusion; it occurs in about 30% of the population. In order to correct a Skeletal Class II Malocclusion in growing patients, functional appliances are commonly used , as they produce a forward movement of the mandible, thus stimulating sagittal mandibular growth [6, 7]. Functional appliances comprehend different types of removable and fixed devices, that are designed to alter the position of the mandible, both sagittally and vertically, to induce supplementary lengthening of the mandible by stimulating increased growth at the condylar cartilage [8-10]. The correction of Class II malocclusion is one of the most common clinical problem interesting the orthodontist, with an estimated one-third of all orthodontic patients treated for this type of malocclusion. It is well known, however, that Class II malocclusion is not a single diagnostic entity but, rather, can result from various skeletal and dento-alveolar components [11, 12]. It would be better to consider a spectrum of Class II malocclusion, and for this reason treatment plan should be done considering the specific characteristics of each patient, and not based on the individual preference of the clinician. Many strategies are available for Class II treatment, and orthodontists have to choose a treatment protocol considering the craniofacial skeleton they believe the appliance will affect the most. For example, Herbst appliance is used to treat patients with a mandibular skeletal retrusion, instead the extra-oral traction that is typically used in patients with a maxillary protrusion. Perhaps more than any other type of functional appliance, whether fixed or removable, the treatment effects induced by the Herbst appliance have been well described in the literature, especially by Pancherz and colleagues [13]. The Herbst appliance typically do not require patient cooperation, but it may have some side effects, because of its anchorage on mandibular premolars that can produce mesial migration of the canines and incisors protrusion. During insertion of the Herbst appliance, the mandible is jumped anteriorly to an incisor edge to edge position with no occlusal contacts present in the posterior dental arch segments; EMG investigation studies demonstrated that after treatment with Herbst appliance, the activity from the masseter e temporal muscles during maximal biting and chewing was markedly increased, especially for the temporal muscle [14]. However, the possibility to achieve an effective treatment results in the absence of patient cooperation, entail that functional fixed appliance used for the correction of skeletal Class II malocclusion produces the best and more predictable clinical outcomes. Some Randomized Clinical Trials (RCTs) [15] and different meta-analyses including prospective studies [16-18] showed that functional appliances can increase mandibular growth in a statistically significant manner. Nevertheless, this increment seems insufficient to determine a clinically significant effect on the skeletal Class II resolution [19]. However, it has to be considered that the paper included in the meta-analyses studies used traditional latero-lateral cranial x-ray to assess treatment outcomes; it is well known that this type of radiological evaluation is invalidated due to the distorsion of the image and absolutely inaccurate individuation of some anatomical point like the Condilion. The objective of the present therapy note is to demonstrate that the use of no compliance apparatus can provide a good correction of skeletal class II malocclusion.

## 2 MATERIALS AND METHODS

**Fig. (1):**
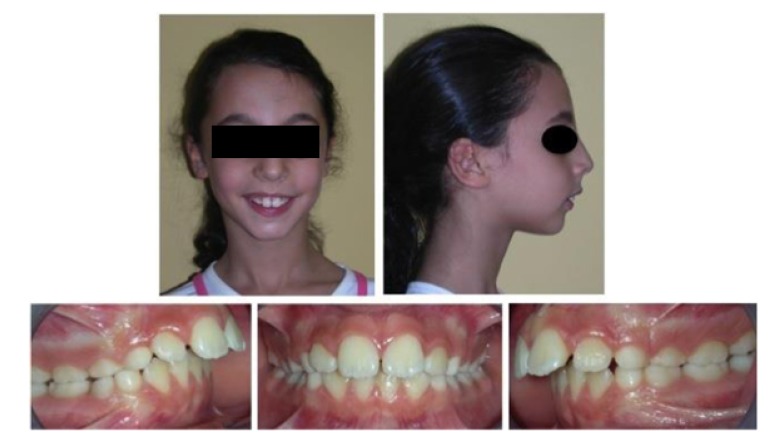
Pre-treatment photographic reports.

In the present study authors reported a therapy note referred to a patient of 10 years old, woman, affected by a skeletal Class II malocclusion, with a severe mandibular retrusion. Patient came in 2013 at the Department of Orthodontics of Messina University for a dental visit; a written informed consent has been acquired from patient’s parents and an orthodontic check-up has been carried out, comprehending intra and extra-oral photos, ortho panoramic and lateral x-rays of the head (Fig. **1**).

**Fig. (2):**
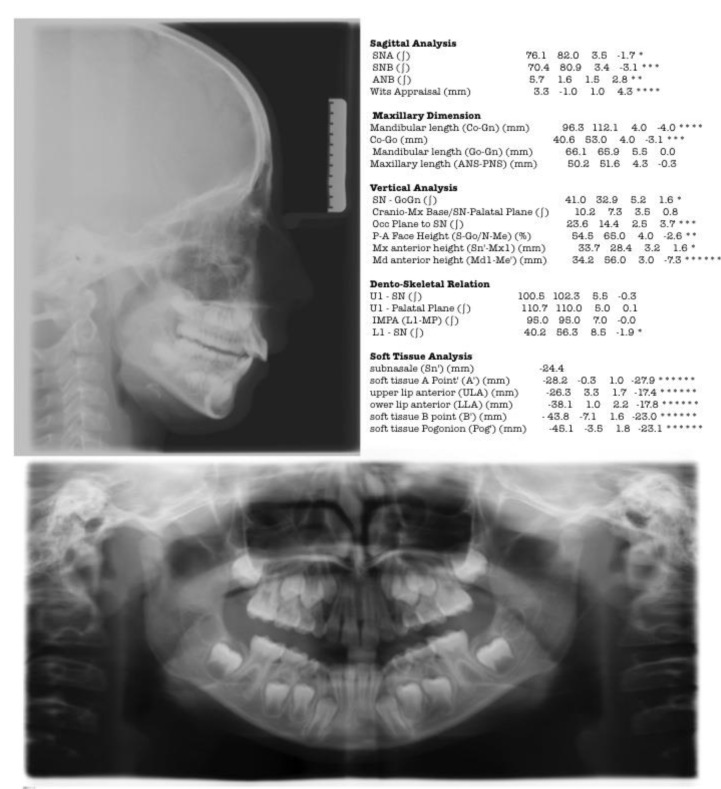
Tele LL and OPT Pre-treatment.

Impressions in alginate of both the arches were taken to obtain dental casts to analyze the occlusal discrepancies. Cephalometric analysis underlined a skeletal Class II malocclusion with a mandibular retrusion, and a low increase of mandibular divergency (Fig. **2**); for this reason, an orthodontic treatment has been planned with the aim of stimulate sagittal mandibular growth.

**Fig. (3):**
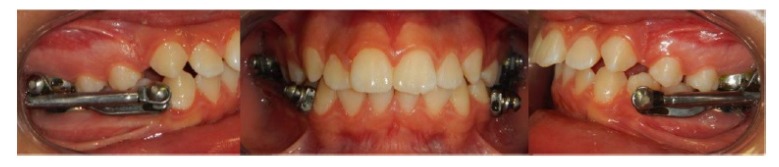
Intra-oral view with Herbst appliance.

In order to stimulate mandibular growth has been used as a Herbst appliance soldered on the bands of first maxillary and mandibular molars with a cantilever design [20]. (American Orthodontics, Sheboygan USA) (Fig. **3**).

**Fig. (4):**
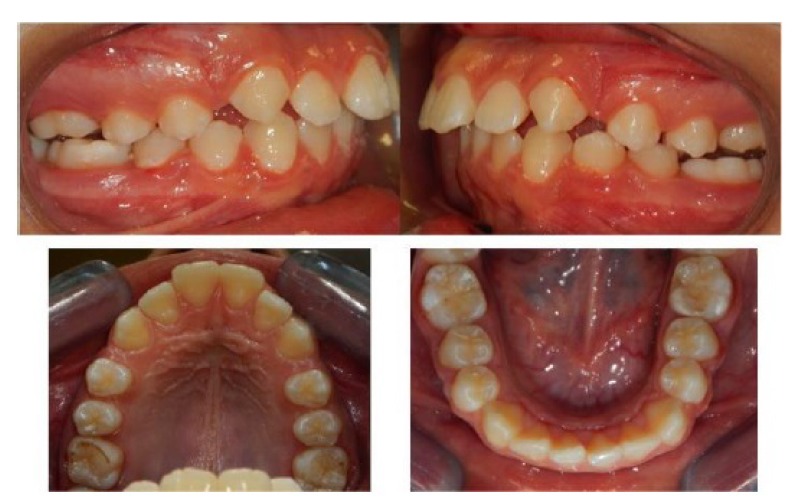
Intra-oral view after Herbst appliance.

After eleven months of therapy with Herbst appliance a bilateral molar class I has been obtained; patients completed the exchange of all deciduous teeth (Fig. **4**), so it has been decided to finalize the occlusion with an orthodontic multibrackets appliance.

**Fig. (5):**
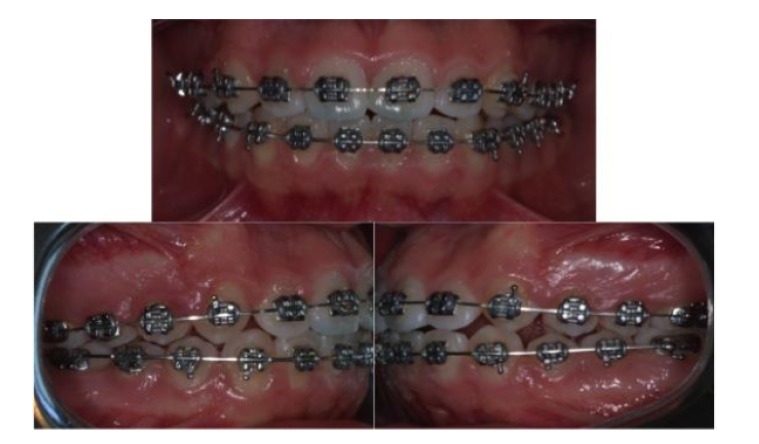
Orthodontic multibrackets appliance.

Considering the low score of dental crowding index, it has been decided to use a traditional twin brackets, Mini Master Serie LP with an MBT prescription (American Orthodontics, Sheboygan USA) (Fig. **5**), and not a self-ligating one. The following arch wire sequence has been used for the present case in both arches:

- 0.014 NiTi SE
- 0.017x0.025 NiTi SE
- 0.019x0.025 NiTi SE
- 0.019x0.025 SS

Class II elastics has been used in order to correct molar class and to reduce maxillary incisor proclination; and at the end of treatment vertical elastics has been used to finalize the occlusion.

After twelve months of treatment, a correct occlusion has been obtained so the multibracket appliance has been removed and a fixed retainer has been applied in the lower arch, instead a Hawley removable appliance has been used for the maintenance in the upper arch.

## 3 RESULTS

**Fig. (6):**
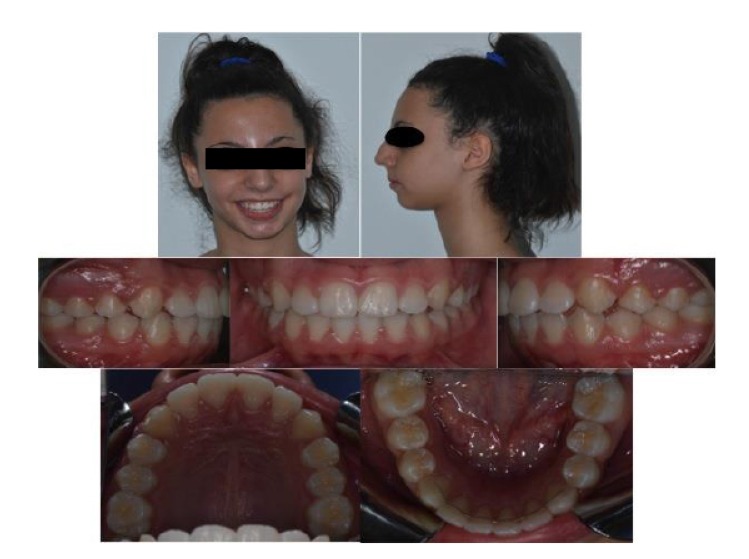
Post-treatment outcomes.

After twenty-three months of therapy with Herbst and multi brackets orthodontic appliance the following treatment outcomes have been achieved: Resolution of mandibular retrusion, resolution of dental crowding in both arches, bilateral molar and canine Class I, correction of overjet and overbite, coincidence of dental midlines, dental occlusal plane correction (Fig. **6**).

**Fig. (7):**
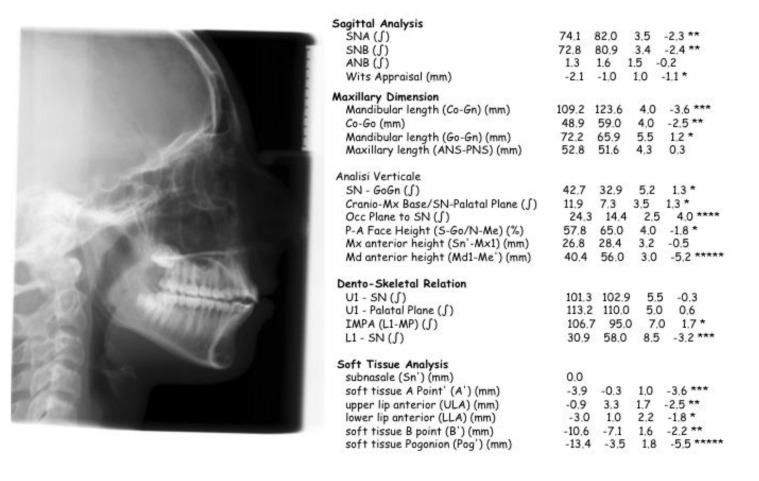
Post-treatment cephalometric analysis.

Post treatment cephalometric analysis demonstrate a significant reduction of ANB angle (- 4.4 °), and an increase in SNB (+2.4°), SN-Go-Gn (+1.7°) and of the occlusal plane to SN (+0.7); this cephalometric outcomes validate the clinical ones and demonstrate that the therapy with Herbst and multibrackets orthodontic appliance produced a better sagittal projection of the mandible, a correction of inter-maxillary relationship and an increase of facial plain divergency (Fig. **7**). The results of cephalometric analysis performed pre and post- treatment has been reported in Table **1**.

## 4 DISCUSSION

The orthodontic treatment reported in the present study showed that the use of noncompliance appliances produces a significant correction of the sagittal discrepancy: this correction is related to different effects such as mandibular growth increasing, glenoid fossa remodeling [5] and lower incisor proclination. The effect of functional appliances on the glenoid fossa is difficult to investigate with traditional lateral cephalometric exams; for a good evaluation is necessary three-dimensional exams such as cone-beam o low dose spiral CT. As a matter of fact, patient compliance can significantly influence treatment outcomes when functional appliances are used for an early correction of Class II malocclusion. Some studies demonstrated that no-compliance appliances are more efficient for the correction of sagittal discrepancy than removable one, because they do not request patient cooperation. Patients compliance in fact is the most unpredictable factor for a good result in the functional therapy of Class II Malocclusion; Herbst appliance led to a normalization of the dento-skeletal patterns without patients’ cooperation. Removable appliance such as the Twin-block appliance seemed to be slightly more efficient in correcting the molar relation-ship and the sagittal maxilla-mandibular skeletal pattern if correctly used, but they request a continuous and constant cooperation by the patients that is so difficult to obtain [21]. In the present study mandibular advancement has been successfully achieved thanks to the use of Herbst appliance, and a good dental relationship has been obtained with the second phase of treatment with an orthodontic multibrackets appliance. Even if the dento-skeletal pattern of the patient before treatment were not ideal for the use of Herbst appliance, we decided to use this type of apparatus anyway, because of the low level of compliance by the patient, who did not want to use a removable appliance, such as an Andresen, that could have controlled better the lower incisor proclination. We managed to maintain good vertical control and to avoid an excessive proclination of the lower incisor. However, a meta-analysis conducted by Marsico *et al*. [17] showed that the functional therapy of II Class malocclusion produces only a small increase in the mandibular length that, even if statistically significant, appear unlikely to be clinically significant. These data seem to support recent reports that 2-phase treatment has no advantages over 1-phase treatment [22]. On the contrary, a systematic review with meta-analysis conducted in 2016 by Zymperdikas *et al*. [23] to assess the treatment effects of fixed functional appliances in treated *versus* untreated Class II patients by means of lateral cephalometric radiographs, fixed functional appliances seem to be effective in improving Class II malocclusion in the short term, even if their effects seem to be mainly dento-alveolar rather than skeletal. However, several benefits must be attributed to the early treatment of Class II malocclusion with functional appliances: prevention of trauma to maxillary incisors associated to an increased overjet, interception of the development of dysfunction, correction of altered neuromuscular patterns, psychosocial advantages for the child during an important formative period of life; is well known in fact that malocclusions and dental anomalies [24] can produce uneasy social relationship in young patients. Early treatment of Class II malocclusion with functional appliances provide also stable dento-alveolar correction, improved prognosis and shorter duration of treatment with fixed appliances. A systematic review published in 2013 by Thiruvenkatachari *et al*. [25] suggests that, providing early orthodontic treatment for children with prominent upper front teeth is more effective in reducing the incidence of incisal trauma than providing the orthodontic treatment when the child is in early adolescence. A multicenter RCT conduced by O’Brien *et al*. [26] showed that early treatment of Class II malocclusion with functional appliances resulted in a correction of overjet and molar class relationships reducing the severity of malocclusion. Most of this correction was due to dento-alveolar changes, but some was due to favorable skeletal corrections. According to this study, early treatment with functional appliances seems to be effective in reducing overjet and severity of malocclusion. The RCT showed that early treatment of Class II malocclusion with functional appliances produce an increase in self-concept and a reduction in negative social experiences [27]. The subjects also reported treatment benefits that could be related to improved self-esteem.

## CONCLUSION

Within the limitations of the present study, according to the results of the therapy note proposed, the following conclusions can be drawn:

- Herbst appliance seems to be efficient in the correction of II Class Malocclusion, independently from patients’ cooperation.
- Even if the increase of mandibular length has been resulted statistically significant but non relevant from a clinical point of view, functional therapy produces an improvement of II Class malocclusion.
- Class II malocclusion correction can be related also to a remodeling of the glenoid fossa and a proclination of lower incisors.
- Even if some studies reports that 2-phase treatment has no advantages over 1-phase treatment, the early correction of Class II malocclusion with functional appliances produce a lot of clinical advantages.

## Figures and Tables

**Table 1:** Cephalometric items Pre and Post-treatment.

Measurement	Pre-Treatment	Post-Treatment
SNA	76.1	74.1
SNB	70.4	72.8
ANB	5.7	1.3
Wits Appraisal (mm)	3.3	-2.1
Condilion-Gnation	96.3	109.2
Condilion-Gonion	40.6	48.9
Gonion-Gnation	66.1	72.2
SNP-SNA	50.2	52.8
SN-GoGn	41.0	42.7
CranioMXBase-SN PalPlane	10.2	11.9
Occl Plane-SN	23.6	24.3
PA Fac Height	54.5	57.8
Max Ant Height	33.7	26.8
Mand Ant Height	34.2	40.4
U1-SN	100.5	101.3
U1-Palatal plane	110.7	113.2
L1-MP	95.0	106.7
L1-SN	40.2	30.9

## References

[1] McLain J.B., Proffitt W.R. (1985). Oral health status in the united states: Prevalence of malocclusion.. J. Dent. Educ..

[2] Proffit W.R., Fields H.W., Moray L.J. (1998). Prevalence of malocclusion and orthodontic treatment need in the United States: Estimates from the NHANES III survey.. Int. J. Adult Orthodon. Orthognath. Surg..

[3] McNamara J.A. (1981). Components of class II malocclusion in children 8-10 years of age.. Angle Orthod..

[4] Agronin K.J., Kokich V.G. (1987). Displacement of the glenoid fossa: A cephalometric evaluation of growth during treatment.. Am. J. Orthod. Dentofacial Orthop..

[5] Portelli M., Gatto E., Matarese G., Militi A., Catalfamo L., Gherlone E., Lucchese A. (2015). Unilateral condylar hyperplasia: Diagnosis, clinical aspects and operative treatment. A case report.. Eur. J. Paediatr. Dent..

[6] Bennet J.C. (2006). Orthodontic management of uncrowded Class II division one malocclusion in children..

[7] Clark W.J. (1988). The twin block technique. A functional orthopedic appliance system.. Am. J. Orthod. Dentofacial Orthop..

[8] Wieslander L., Lagerström L. (1979). The effect of activator treatment on class II malocclusions.. Am. J. Orthod..

[9] Moss M.L., Salentijn L. (1969). The primary role of functional matrices in facial growth.. Am. J. Orthod..

[10] Fränkel R. (1969). The treatment of Class II, Division 1 malocclusion with functional correctors.. Am. J. Orthod..

[11] Moyers R.E., Riolo M.L., Guire K.E., Wainright R.L., Bookstein F.L. (1980). Differential diagnosis of class II malocclusions. Part 1. Facial types associated with class II malocclusions.. Am. J. Orthod..

[12] Cordasco G., Portelli M., Militi A., Nucera R., Lo Giudice A., Gatto E., Lucchese A. (2013). Low-dose protocol of the spiral CT in orthodontics: Comparative evaluation of entrance skin dose with traditional X-ray techniques.. Prog. Orthod..

[13] Hägg U., Pancherz H. (1988). Dentofacial orthopaedics in relation to chronological age, growth period and skeletal development. An analysis of 72 male patients with class II division 1 malocclusion treated with the herbst appliance.. Eur. J. Orthod..

[14] Pancherz H., Anehus-Pancherz M. (1980). Muscle activity in class II, division 1 malocclusions treated by bite jumping with the Herbst appliance. An electromyographic study.. Am. J. Orthod..

[15] Tulloch J.F., Phillips C., Koch G., Proffit W.R. (1997). The effect of early intervention on skeletal pattern in class II malocclusion: A randomized clinical trial.. Am. J. Orthod. Dentofacial Orthop..

[16] O’Brien K., Wright J., Conboy F., Sanjie Y., Mandall N., Chadwick S., Connolly I., Cook P., Birnie D., Hammond M., Harradine N., Lewis D., McDade C., Mitchell L., Murray A., O’Neill J., Read M., Robinson S., Roberts-Harry D., Sandler J., Shaw I. (2003). Effectiveness of early orthodontic treatment with the twin-block appliance: A multicenter, randomized, controlled trial. Part 1: Dental and skeletal effects.. Am. J. Orthod. Dentofacial Orthop..

[17] Marsico E., Gatto E., Burrascano M., Matarese G., Cordasco G. (2011). Effectiveness of orthodontic treatment with functional appliances on mandibular growth in the short term.. Am. J. Orthod. Dentofacial Orthop..

[18] Antonarakis G.S., Kiliaridis S. (2007). Short-term anteroposterior treatment effects of functional appliances and extraoral traction on class II malocclusion. A meta-analysis.. Angle Orthod..

[19] Koretsi V., Zymperdikas V.F., Papageorgiou S.N., Papadopoulos M.A. (2015). Treatment effects of removable functional appliances in patients with class II malocclusion: A systematic review and meta-analysis.. Eur. J. Orthod..

[20] Lucchese A., Carinci F., Brunelli G., Monguzzi R. (2011). An *in vitro* study of resistance to corrosion in brazed and laser-welded orthodontic appliances.. Eur. J. Inflamm..

[21] Tulloch J.F., Proffit W.R., Phillips C. (2004). Outcomes in a 2-phase randomized clinical trial of early class II treatment.. Am. J. Orthod. Dentofacial Orthop..

[22] Harrison JE, O’Brien KD, Worthington HV

[23] Zymperdikas V.F., Koretsi V., Papageorgiou S.N., Papadopoulos M.A. (2016). Treatment effects of fixed functional appliances in patients with Class II malocclusion: A systematic review and meta-analysis.. Eur. J. Orthod..

[24] Militi D., Militi A., Cutrupi M.C., Portelli M., Rigoli L., Matarese G., Salpietro D.C. (2011). Genetic basis of non syndromic hypodontia: A DNA investigation performed on three couples of monozygotic twins about PAX9 mutation.. Eur. J. Paediatr. Dent..

[25] Thiruvenkatachari B., Harrison J.E., Worthington H.V., O’Brien K.D. (2013). Orthodontic treatment for prominent upper front teeth (Class II malocclusion) in children.. Cochrane Database Syst. Rev..

[26] O’Brien K., Wright J., Conboy F., Sanjie Y., Mandall N., Chadwick S., Connolly I., Cook P., Birnie D., Hammond M., Harradine N., Lewis D., McDade C., Mitchell L., Murray A., O’Neill J., Read M., Robinson S., Roberts-Harry D., Sandler J., Shaw I. (2003). Effectiveness of early orthodontic treatment with the twin-block appliance: A multicenter, randomized, controlled trial. Part 1: Dental and skeletal effects.. Am. J. Orthod. Dentofacial Orthop..

[27] O’Brien K., Wright J., Conboy F., Chadwick S., Connolly I., Cook P., Birnie D., Hammond M., Harradine N., Lewis D., McDade C., Mitchell L., Murray A., O’Neill J., Read M., Robinson S., Roberts-Harry D., Sandler J., Shaw I., Berk N.W. (2003). Effectiveness of early orthodontic treatment with the Twin-block appliance: A multicenter, randomized, controlled trial. Part 2: Psychosocial effects.. Am. J. Orthod. Dentofacial Orthop..

